# Supplementary material for: Biomonitoring of benzophenones in guano samples of wild bats in Poland
Source: PLoS One. 2024 Apr 9;19(4):e0301727. doi: 10.1371/journal.pone.0301727 (PMC11003676; doi:10.1371/journal.pone.0301727)
Supplement: S1 File — (DOCX) [file pone.0301727.s001.docx]

**SUPPLEMENTARY MATERIALS to:**

**Biomonitoring benzophenones in guano samples of wild bats in Poland**

Slawomir Gonkowski^1^, Julia Martín^2^, Irene Aparicio^2^, Juan Luis Santos^2^, Esteban Alonso^2^, Liliana Rytel^3^

^1^Department of Clinical Physiology, Faculty of Veterinary Medicine, University of Warmia and Mazury, Street Oczapowskiego 14, 10-719 Olsztyn, Poland.

^2^Departamento de Química Analítica, Universidad de Sevilla, C/ Virgen de África, 7, E-41011, Sevilla, Spain.

^3^Department of Internal Diseases with Clinic, Faculty of Veterinary Medicine, University of Warmia and Mazury in Olsztyn, ul. Oczapowskiego 14, 10-719, Olsztyn, Poland.

| Compound | Internal standard | MRM1 | MRM2 | Fragmentator (V) | Collision Energy (eV) | Ionization mode |
| --- | --- | --- | --- | --- | --- | --- |
| BP-1 | BP-d_10_ | 215>137 | 215>105 | 160 | 16 | ESI+ |
| BP-2 | BP-d_10_ | 245>135 | 245>109 | 160 | 12 | ESI- |
| BP-3 | BP-d_10_ | 229>151.1 | 229>105 | 160 | 16 | ESI+ |
| BP-8 | BP-d_10_ | 245>121 | 245>65 | 160 | 16 | ESI+ |

Table S1. MRM conditions used for LC-MS/MS of benzophenones (BPs)

MRM 1: transition used for quantification; MRM 2: transition used for confirmation**.**

Table S2. Linearity, method detection limit (MDL), method quantification limit (MQL), precision (relative standard deviation, RSD) and recovery (Rec) of BPs in guano matrix

| Compound | Linearity  R^2^ | MDL  (ng/g dw) | MQL  (ng/g dw) | Rec  % | RSD  % |
| --- | --- | --- | --- | --- | --- |
| BP-1 | 0.999 | 0.04 | 0.10 | 107.7 | 18.6 |
| BP-2 | 0.990 | 0.04 | 0.10 | 120.3 | 13.5 |
| BP-3 | 0.996 | 0.04 | 0.10 | 111.9 | 14.0 |
| BP-8 | 0.999 | 0.04 | 0.10 | 102.1 | 9.2 |

Table S3. Concentration levels (ng/g dw) of BPs in bat guano samples

| Bat colony no. | Sample no. | BP-1 | BP-2 | BP-3 | BP-8 |
| --- | --- | --- | --- | --- | --- |
| 1 | 1 | 10.5 | <MDL | <MQL | <MDL |
|  | 2 | 13.2 | <MDL | <MQL | <MDL |
|  | 3 | 13.7 | <MDL | <MQL | <MDL |
|  | 4 | 20.8 | <MDL | <MQL | <MDL |
|  | 5 | 34.8 | <MDL | 15.5 | <MDL |
|  | 6 | 18.6 | <MDL | 0.84 | <MDL |
|  | 7 | 18.7 | <MDL | 2.79 | <MDL |
|  | 8 | 13.2 | <MDL | <MQL | <MDL |
|  | 9 | 20.1 | <MDL | 1.43 | <MDL |
|  | 10 | 17.2 | <MDL | 4.61 | <MDL |
| 2 | 1 | 78.4 | <MDL | <MQL | <MDL |
|  | 2 | 259 | <MDL | 14.6 | <MDL |
|  | 3 | 63.7 | <MDL | <MQL | <MDL |
|  | 4 | 30.5 | <MDL | <MQL | <MDL |
|  | 5 | 67.3 | <MDL | <MQL | <MDL |
|  | 6 | 50.1 | <MDL | <MQL | <MDL |
|  | 7 | 68.0 | <MDL | 1.44 | <MDL |
|  | 8 | 52.9 | <MDL | <MQL | <MDL |
|  | 9 | 43.1 | <MDL | <MQL | <MDL |
|  | 10 | 54.1 | <MDL | <MQL | <MDL |
| 3 | 1 | 58.7 | <MDL | 0.44 | <MDL |
|  | 2 | 62.8 | <MDL | 15.2 | <MDL |
|  | 3 | 57.0 | <MDL | 5.50 | <MDL |
|  | 4 | 48.6 | <MDL | 5.48 | <MDL |
|  | 5 | 57.2 | <MDL | <MQL | <MDL |
|  | 6 | 34.4 | <MDL | <MQL | <MDL |
|  | 7 | 41.5 | <MDL | <MQL | <MDL |
|  | 8 | 66.9 | <MDL | <MQL | <MDL |
|  | 9 | 55.4 | <MDL | 2.19 | <MDL |
|  | 10 | 87.2 | <MDL | <MQL | <MDL |
| 4 | 1 | 45.6 | <MDL | 6.67 | <MDL |
|  | 2 | 5.09 | <MDL | 10.3 | <MDL |
|  | 3 | 6.34 | <MDL | <MQL | <MDL |
|  | 4 | 7.99 | <MDL | <MQL | <MDL |
|  | 5 | 10.6 | <MDL | <MQL | <MDL |
|  | 6 | 4.03 | <MDL | <MQL | <MDL |
|  | 7 | <MQL | <MDL | <MQL | <MDL |
|  | 8 | 10.9 | <MDL | 19.0 | <MDL |
|  | 9 | 6.33 | <MDL | 0.73 | <MDL |
|  | 10 | 4.16 | <MDL | <MQL | <MDL |

Compound acronyms: BP-1: benzophenone 1; BP-2: benzophenone 2; BP-3: benzophenone 3; BP-8 benzophenone 8; <MQL: Below Method Quantification Limit (0.10 ng/g dw); <MDL: Below Method Detection Limit (0.04 ng/g dw)
