# Supplementary material for: Biomonitoring of benzophenones in guano samples of wild bats in Poland
Source: PLoS One. 2024 Apr 9;19(4):e0301727. doi: 10.1371/journal.pone.0301727 (PMC11003676; doi:10.1371/journal.pone.0301727)
Supplement: Attachment — Submitted filename: Reviewer Attachments PONE-D-23-43946. PSMA.docx [file pone.0301727.s002.docx]

PLOS ONE

# Biomonitoring benzophenones in guano samples of wild bats in Poland

--Manuscript Draft--

| Manuscript Number: | PONE-D-23-43946 |
| --- | --- |
| Article Type: | Research Article |
| Full Title: | Biomonitoring benzophenones in guano samples of wild bats in Poland |
| Short Title: | Benzophenones in wild bats in Poland |
| Corresponding Author: | Liliana Rytel  University of Warmia and Mazury in Olsztyn Faculty of Veterinary Medicine: Uniwersytet Warminsko-Mazurski w Olsztynie Wydzial Medycyny Weterynaryjnej Olsztyn, POLAND |
| Keywords: | benzophenone, wild animals, exposure, feces, environment pollution, wildlife |
| Abstract: | Benzophenones (BPs) are substances used to produce sunscreens, cosmetics and personal care products. The knowledge on BPs in wild terrestrial mammals is practically lacking. Therefore, the aim of the study was to assess the concentration levels of selected BPs commonly used in the industry including benzophenone 1 (BP- 1), benzophenone 2 (BP-2), benzophenone 3 (BP-3) and benzophenone 8 (BP-8) in guano samples collected from colonies of greater mouse eared bats (Myotis myotis) using liquid chromatography with the tandem mass spectrometry (LC-MS/MS) method. Concentration levels of BP-1 above method quantification limit (MQL) were noted in 97.5% samples and fluctuated from <0.1 ng/g to 259 ng/g with a mean value of 41.50 ng/g. BP-3 concentration levels higher than MQL were observed in 40% of samples, and fluctuated from <0.1 ng/g to 19 ng/g with a mean value of 6.67 ng/g. BP-2 and  BP-8 concentration levels did not exceed the method detection limit (0.04 ng/g) in any sample included in the analysis. The results show that such samples are a good alternative to “classic” matrices (urine or blood) in the assessment of the exposure to BPs, especially in protected wild animals, where great importance should be attached to not disturbing and stressing the animals. |
| Order of Authors: | Slawomir Gonkowski |
|  | Julia Martin |
|  | Irene Aparicio |
|  | Juan Luis Santos |
|  | Esteban Alonso |
|  | Andrzej Pomianowski |
|  | László Könyves |
|  | Liliana Rytel |
| Additional Information: |  |
| Question | Response |
| Financial Disclosure  Enter a financial disclosure statement that describes the sources of funding for the work included in this submission. Review the [submission guidelines](http://journals.plos.org/plosone/s/submission-guidelines#loc-financial-disclosure-statement) for detailed requirements. View published research articles from [*PLOS ONE*](https://journals.plos.org/plosone/) for specific examples. | The author(s) received no specific funding for this work. |

| This statement is required for submission and will appear in the published article if the submission is accepted. Please make sure it is accurate. |  |
| --- | --- |
| Funded studies  Enter a statement with the following details:   - Initials of the authors who received each award - Grant numbers awarded to each author - The full name of each funder - URL of each funder website - Did the sponsors or funders play any role in the study design, data collection and analysis, decision to publish, or preparation of the manuscript? |  |
| Did you receive funding for this work? |  |
| Competing Interests  Use the instructions below to enter a competing interest statement for this submission. On behalf of all authors, disclose any [competing interests](http://journals.plos.org/plosone/s/competing-interests) that could be perceived to bias this work—acknowledging all financial support and any other relevant financial or non- financial competing interests.  This statement is required for submission and will appear in the published article if the submission is accepted. Please make sure it is accurate and that any funding sources listed in your Funding Information later in the submission form are also declared in your Financial Disclosure statement.  View published research articles from  [*PLOS ONE*](http://journals.plos.org/plosone/) for specific examples. | The authors have declared that no competing interests exist. |

| NO authors have competing interests  Enter: *The authors have declared that no competing interests exist.*  Authors with competing interests  Enter competing interest details beginning with this statement:  *I have read the journal's policy and the authors of this manuscript have the following competing interests: [insert competing interests here]* |  |
| --- | --- |
| * typeset |  |
| Ethics Statement  Enter an ethics statement for this submission. This statement is required if the study involved:   - Human participants - Human specimens or tissue - Vertebrate animals or cephalopods - Vertebrate embryos or tissues - Field research   Write "N/A" if the submission does not require an ethics statement.  General guidance is provided below. Consult the [submission guidelines](http://journals.plos.org/plosone/s/submission-guidelines#loc-guidelines-for-specific-study-types) for detailed instructions. Make sure that all information entered here is included in the Methods section of the manuscript. | Not applicable. Due to the fact that sampling was not invasive to animals, in view of the Act for the Protection of Animals for Scientific or Educational Purposes of 15 January 2015 (Official Gazette 2015, No. 266), applicable in the Republic of Poland, the agreement of Ethical Committee was not required during this investigation. |

| Format for specific study types  Human Subject Research (involving human participants and/or tissue)   - Give the name of the institutional review board or ethics committee that approved the study - Include the approval number and/or a statement indicating approval of this research - Indicate the form of consent obtained (written/oral) or the reason that consent was not obtained (e.g. the data were analyzed anonymously)   Animal Research (involving vertebrate animals, embryos or tissues)   - Provide the name of the Institutional Animal Care and Use Committee (IACUC) or other relevant ethics board that reviewed the study protocol, and indicate whether they approved this research or granted a formal waiver of ethical approval - Include an approval number if one was obtained - If the study involved *non-human primates*, add *additional details* about animal welfare and steps taken to ameliorate suffering - If anesthesia, euthanasia, or any kind of animal sacrifice is part of the study, include briefly which substances and/or methods were applied   Field Research  Include the following details if this study involves the collection of plant, animal, or other materials from a natural setting:   - Field permit number - Name of the institution or relevant body that granted permission |  |
| --- | --- |
|  |  |
| Data Availability  Authors are required to make all data underlying the findings described fully available, without restriction, and from the time of publication. PLOS allows rare exceptions to address legal and ethical concerns. See the [PLOS Data Policy](http://journals.plos.org/plosone/s/data-availability) and [FAQ](http://journals.plos.org/plosone/s/data-availability#loc-faqs-for-data-policy) for detailed information. | Yes - all data are fully available without restriction |

| A Data Availability Statement describing where the data can be found is required at submission. Your answers to this question constitute the Data Availability Statement and will be published in the article, if accepted. |  |
| --- | --- |
| Important: Stating ‘data available on request from the author’ is not sufficient. If your data are only available upon request, select ‘No’ for the first question and explain your exceptional situation in the text box. |  |
| Do the authors confirm that all data underlying the findings described in their manuscript are fully available without restriction? |  |
| Describe where the data may be found in full sentences. If you are copying our sample text, replace any instances of XXX with the appropriate details.   - If the data are held or will be held in a public repository, include URLs, accession numbers or DOIs. If this information will only be available after acceptance, indicate this by ticking the box below. For example: *All XXX files are available from the XXX database (accession number(s) XXX, XXX.*). - If the data are all contained within the manuscript and/or Supporting Information files, enter the following: *All relevant data are within the manuscript and its Supporting Information files.* - If neither of these applies but you are able to provide details of access elsewhere, with or without limitations, please do so. For example:   *Data cannot be shared publicly because of [XXX]. Data are available from the XXX Institutional Data Access / Ethics Committee (contact via XXX) for researchers who meet the criteria for access to confidential data.*  *The data underlying the results presented in the study are available from (include the name of the third party* | All data is included in the manuscript and the supplementary materials. |

| *and contact information or URL).*   - This text is appropriate if the data are owned by a third party and authors do not have permission to share the data.   * typeset |  |
| --- | --- |
| Additional data availability information: |  |

Manuscript	[Click here to access/download;Manuscript;Manuscript Plos](https://www.editorialmanager.com/pone/download.aspx?id=35060536&guid=dc963215-0bef-4547-ae7b-b4502beb52b7&scheme=1)[<https://www.editorialmanager.com/pone/download.aspx?id=35060536&guid=dc963215-0bef-4547-ae7b-b4502beb52b7&scheme=1> One.docx](https://www.editorialmanager.com/pone/download.aspx?id=35060536&guid=dc963215-0bef-4547-ae7b-b4502beb52b7&scheme=1)

## 1 Biomonitoring benzophenones in guano samples of wild bats in Poland

2 **Benzophenones in wild bats in Poland**
3 [Slawomir Gonkowski](https://pubmed.ncbi.nlm.nih.gov/?term=Gonkowski%20S%5BAuthor%5D)^1^, [Julia Martín](https://pubmed.ncbi.nlm.nih.gov/?term=Martín%20J%5BAuthor%5D)^2^, [Irene Aparicio](https://pubmed.ncbi.nlm.nih.gov/?term=Aparicio%20I%5BAuthor%5D)^2^, [Juan Luis Santos](https://pubmed.ncbi.nlm.nih.gov/?term=Santos%20JL%5BAuthor%5D)^2^, [Esteban](https://pubmed.ncbi.nlm.nih.gov/?term=Alonso%20E%5BAuthor%5D)
4 [Alonso](https://pubmed.ncbi.nlm.nih.gov/?term=Alonso%20E%5BAuthor%5D)^2^, Andrzej Pomianowski^3^ László Könyves^4^, and [Liliana Rytel](https://pubmed.ncbi.nlm.nih.gov/?term=Rytel%20L%5BAuthor%5D)^3^
5 ^1^Department of Clinical Physiology, Faculty of Veterinary Medicine, University of
6 Warmia and Mazury, Oczapowskiego Str. 14, 10-719 Olsztyn, Poland
7 ^2^Departamento de Química Analítica, Universidad de Sevilla, C/Virgen de África, 7, E-
8 41011 Sevilla, Spain
9 ^3^Department of Internal Diseases with Clinic, Faculty of Veterinary Medicine,
10 University of Warmia and Mazury in Olsztyn, Oczapowskiego Str. 14, 10-719 Olsztyn, Poland
11 ^4^Department of Animal Hygiene, Herd Health and Mobile Clinic, University of
12 Veterinary Medicine, 1078 Budapest, Hungary
13 ^*^Correspondence: Liliana Rytel, [Liliana.rytel@uwm.edu.pl](mailto:Liliana.rytel@uwm.edu.pl)

## 14 Abstract

15 Benzophenones (BPs) are substances used to produce sunscreens, cosmetics and
16 personal care products. The knowledge on BPs in wild terrestrial mammals is practically
17 lacking. Therefore, the aim of the study was to assess the concentration levels of selected BPs
18 commonly used in the industry including benzophenone 1 (BP-1), benzophenone 2 (BP-2),
19 benzophenone 3 (BP-3) and benzophenone 8 (BP-8) in guano samples collected from colonies
20 of greater mouse eared bats **(***Myotis myotis*) using liquid chromatography with the tandem mass
21 spectrometry (LC-MS/MS) method. Concentration levels of BP-1 above method quantification
22 limit (MQL) were noted in 97.5% samples and fluctuated from <0.1 ng/g to 259 ng/g with a
23 mean value of 41.50 ng/g. BP-3 concentration levels higher than MQL were observed in 40%
24 of samples, and fluctuated from <0.1 ng/g to 19 ng/g with a mean value of 6.67 ng/g. BP-2 and
25 BP-8 concentration levels did not exceed the method detection limit (0.04 ng/g) in any sample
26 included in the analysis. The results show that such samples are a good alternative to “classic”
27 matrices (urine or blood) in the assessment of the exposure to BPs, especially in protected wild
28 animals, where great importance should be attached to not disturbing and stressing the animals.

## 29 Keywords

30 benzophenone, wild animals, exposure, feces, environment pollution, wildlife

## 31 Introduction

32 Benzophenones (BPs) are a group of substances consisting on two phenyl groups linked
33 to a carbonyl group (Gavrila et al. 2023). Some of the BPs are of natural origin and are mainly
34 produced by fungi and plants (Wu et al. 2014). Some of them are synthesized for various
35 branches of industry. [It is estimated that](https://context.reverso.net/tłumaczenie/angielski-polski/it%2Bis%2Bestimated%2Bthat) 10,000 tons of BPs are produced annually (Song et al.
36 2020). BPs absorb ultraviolet (UV) radiation and therefore first of all they [are used for the](https://context.reverso.net/tłumaczenie/angielski-polski/are%2Bused%2Bfor%2Bthe%2Bproduction)
37 [production](https://context.reverso.net/tłumaczenie/angielski-polski/are%2Bused%2Bfor%2Bthe%2Bproduction) of sunscreens with UV filters, cosmetics, flavorings or perfumes, hair dyes,
38 shampoos, and personal care products (Vila et al. 2015; Foubert et al. 2021). Moreover, BPs
39 are part of the food containers, bottles, paints, inks, plastics, car polishes, fabrics, and many
40 other items, where they perform the functions of UV stabilizers (Liao and Kannan 2014; Xue
41 et al. 2017; Gavrila et al. 2023). There are 12 main different benzophenone derivatives, among
42 which	the	most	commonly	used	in	industry	are	benzophenone	1	(BP-1,	4-
43 dihydroxybenzophenone),	benzophenone	2	(BP-2,	2,2',4,4'-tetrehydroxybenzophenone),
44 benzophenone 3 (BP-3, 2-hydroxy-4-methoxybenzophenone, oxybenzone) and benzophenone
45 8 (BP-8, dioxybenzone**)** (Song et al. 2020).
46 Due to their widespread use, BPs are increasingly reaching the natural environment. The
47 environmental pollution by BPs is favored by their chemical properties, namely high stability,
48 resistance to UV radiation, and ability to accumulate in water (Mao et al. 2019). Previous
49 studies have reported the presence of BPs, mainly in the surface inland and oceanic water (Ma
50 et al. 2016; Tang et al. 2018; Dulsat-Masvidal et al. 2023). BPs also pollute tap water, air and
51 soil, especially agricultural soils treated with compost from sewage sludge (Zhang et al. 2011;
52 Careghini et al. 2015; Sun et al. 2015). These substances may also penetrate to plants, which
53 [are food](https://context.reverso.net/tłumaczenie/angielski-polski/are%2Bfood) for humans and animals (Careghini et al. 2015; Camino-Sánchez et al. 2016; Aparicio
54 et al. 2018). The concentration levels of BPs clearly depend on the urbanization and
55 industrialization of region, where studies have been conducted, as well as on human activity in
56 a given area (Kim and Choi 2014; Careghini et al. 2015; Schneider and Lim 2019).
57 The presence of BPs in various elements of the environment results in the exposure of
58 humans and animals to these substances, which may get inside the living organisms through the
59 digestive tract, skin, and respiratory system, as well as through the placenta during prenatal
60 period (Kadry et al. 1995; Wan et al. 2015; Hewitt et al. 2020; Song et al. 2020; Holmboe et al.
61 2022).
62 Exposure to BPs can cause disturbances in the functions of many internal organs. [This](https://context.reverso.net/tłumaczenie/angielski-polski/This%2Bis%2Bbecause)
63 [is because](https://context.reverso.net/tłumaczenie/angielski-polski/This%2Bis%2Bbecause) BPs may bind to receptors of various hormones, including estrogen, androgen, and
64 thyroid hormone, and therefore show endocrine disrupting properties, which depend on the type
65 of BP, dose and animal species or type of cells, on which BPs act (Kim and Choi 2014).
66 Furthermore, BPs show genotoxic and mutagenic properties (Wnuk et al. 2022). It is known
67 that BPs affect, among others, male, and female reproductive system, where exposure to these
68 compounds results in perturbation in sperm development, decrease in the number of oocytes,
69 elongation of the estrus cycle and disorders in the early germ cell development (Nakamura et
70 al. 2015; Santamaria et al. 2019; Wnuk et al. 2022). Some studies have also described
71 connections between BPs and infertility and the risk of endometriosis (Kunisue et al. 2012;
72 Buck et al. 2014). In turn, within the nervous system, BPs cause a number of changes, including,
73 among others, alterations in the epigenetic state of neuronal cells, mitochondrial membrane
74 potentials, and various levels of neuronal factors, which in turn can lead to abnormal brain
75 development and an increased risk of neurodegenerative diseases (Wnuk et al. 2019, 2022).
76 Some studies have found that BPs have an obesogenic activity (Shin et al. 2020).
77 Due to mentioned above adverse effects of BPs, it is important to monitor the exposure
78 to these substances in humans and animals. The vast majority of previous publications have
79 been related to exposure of BPs in humans, where the presence of these compounds has been
80 confirmed in various types of samples (Mao et al. 2022). BPs have been described not only in
81 typical matrices such as urine (Rocha et al. 2018; Wang et al. 2021) and blood - whole blood,
82 serum, and plasma (Li et al. 2019; Song et al. 2020; Frederiksen et al. 2021), but also among
83 others in amniotic fluid (Song et al. 2020), seminal plasma (Frederiksen et al. 2021), placenta
84 (Vela-Soria et al. 2011) and breast milk (Molins-Delgado et al. 2018a).
85 Contrary to humans, knowledge about wild animal exposure to BPs is relatively limited.
86 Previous studies mainly describe the concentration levels of BPs in aquatic organisms such as
87 marine mammals (Blouin et al. 2022), turtles (Cocci et al. 2020), fish (Balmer et al. 2005; Kim
88 and Choi 2014), mussels (Castro et al. 2022), prawns, and urchins (Sang and Leung 2016). In
89 addition, BPs have also been found in the eggs and internal organs of wild marine and terrestrial
90 birds (Molins-Delgado et al. 2017; González-Rubio et al. 2020; Oró-Nolla et al. 2021).
91 To the best knowledge of the authors, there are no studies on the exposure of terrestrial
92 wild mammals to BPs with the exception of our earlier methodological publication describing
93 the methodology of examining bat guano for the presence of BPs on a very limited number of
94 individuals (n=5) (Martin et al., 2023).
95 However, it is very likely that terrestrial animals are exposed to BPs present in different
96 elements of the natural environment. One of group of animals, which play important role in
97 the ecotoxicology are bats. It is a large group of animals of over 1400 species living [in different](https://context.reverso.net/tłumaczenie/angielski-polski/in%2Bdifferent%2Bclimatic%2Bzones)
98 [climatic zones](https://context.reverso.net/tłumaczenie/angielski-polski/in%2Bdifferent%2Bclimatic%2Bzones) (Wilson and Mittermeier 2019). Bats are highly sensitive to environmental
99 pollution and any environmental change, which is the reason for the continuous reduction in
100 their population and the protection of many bat species (Russo and Ancillotto 2015). Due to
101 their sensitivity to environmental factors, bats are often used as a bioindicator of environmental
102 quality during toxicological studies (Russo et al. 2021; Sotero et al. 2022).
103 It should be pointed out that a big problem in studies on wild animal exposure to
104 compounds polluting the environment is a selection of the matrix, in which the levels of the
105 investigated substances are determined. Collection of the majority of matrices using in
106 toxicology, including urine, blood, or hair samples, is practically impossible without capturing
107 the animals, which is a strong stress factor for them and a significant interference with their life
108 and environment. This is particularly important during studies on protected species.
109 Feces/guano samples are practically the only matrix, which may be collected without the need
110 for significant interference in the animal life. Moreover, our previous methodological study has
111 shown that guano samples can be used to determine BPs levels (Martin et al., 2023).
112 Therefore, the aim of the present study was to evaluate the concentration levels of
113 selected BPs commonly used in the industry (BP-1, BP-2, BP-3, BP-8) in guano samples
114 collected from the greater mouse-eared bats (*Myotis myotis*) living in various parts of Poland.
115 This study, which, to the best of our knowledge, is the first investigation on terrestrial mammals
116 to BPs and first use of guano/feces samples to this aim will contribute to expanding knowledge
117 on BPs in wild animals.

## 118 Materials and methods

119 **Reagents**
120 The following reagents were used during the study: formic acid and ammonium acetate
121 from Panreac (Barcelona, Spain); C18 disperser sorbent from Scharlab (Barcelona, Spain);
122 HPLC-grade methanol, and water from Romil (Barcelona, Spain); high purity standards of
123 benzophenone-1 (BP-1), benzophene-2 (BP-2), benzophenone-3 (BP-3), benzophenone-8 (BP-
124 8) from Sigma-Aldrich (Steinheim, Germany). Isotopically labelled BP used as internal
125 standard (BP-d10) was purchased from Cambridge Isotope Laboratories (Tewksbury, MA,
126 USA). Individual stock standard solutions were prepared at 1000 mg/L in methanol and kept at
127 -18^0^C. Working solutions were made up by dilution of the stock standard solution in methanol.

## 128 Sample collection

129 Bat guano samples were collected in August-September 2021 from four summer
130 (nursery) colonies of greater mouse eared bats (*Myotis myotis*) located in various regions of
131 Poland (Figure 1).

7

133 The characterization of bat colonies included into the study is presented in table 1.
134 Table 1 Characterization of localization of bat colonies included into the study

| Bat colony No. | 1 | 2 | 3 | 4 |
| --- | --- | --- | --- | --- |
| Name of place | Brenna | Sliwice | Pulawy | Opole |
|  |  |  |  | Lubelskie |
| Type of place | village | village | City | town |
| Human population | 11285 | 2464 | 47417 | 8605 |
| Population density (per km^2^) | 76.6 | 31.3 | 962.1 | 561.4 |
| Industry | none | none | Chemical, | food |
|  |  |  | pharmaceutical, |  |
|  |  |  | construction, food |  |
| Dominant landform | forest | agriculural | industrial | small-town |
| Distance [in a straight line](https://context.reverso.net/tłumaczenie/angielski-polski/distance%2Bin%2Ba%2Bstraight%2Bline) from the  nearest large urban and industrial | 61 | 66 | 46 | 43 |
| center (over 300,000 inhabitants) |  |  |  |  |
| in km |  |  |  |  |
| Location of bat colony | School attic | Church tower | Children’s home attic | School attic |
| Approximate animal number in colony | 250 | 450 | 250 | 200 |
| coordinates | 49°43'37.4"N  18°53'46.3"E | 53°42'19.0"N  18°10'04.4"E | 51°25'19.9"N  21°58'37.4"E | 51°09'02.7"N  21°58'24.6"E |

135

136 Sampling was done as follows: flat glass containers were placed at different points of
137 the floor in the space, where bat colony was located. After 48 hours, containers were removed
138 and bat guano samples were placed in glass jars, frozen, and stored at -20^º^C for further analysis.
139 Ten guano samples collected from various parts of the colonies of each bat colony studied were
140 included into investigation. All activities during sampling were carried out in such a way that
141 not to scare or stress the animals. Due to the fact that sampling was not invasive to animals, [in](https://context.reverso.net/tłumaczenie/angielski-polski/in%2Bview%2Bof%2Bthe%2Blaw)
142 [view of](https://context.reverso.net/tłumaczenie/angielski-polski/in%2Bview%2Bof%2Bthe%2Blaw) the Act for the Protection of Animals for Scientific or Educational Purposes of 15
143 January 2015 (Official Gazette 2015, No. 266), applicable in the Republic of Poland, the
144 agreement of Ethical Committee was not required during this investigation. The samples were
145 collected and stored in a way that prevents their accidental extrinsic pollution with BPs.

## 146 1.1.1. Sample treatment

147 Sample treatment was carried out according to a method previously described by Martin
148 et al. (2023). Guano samples were lyophilized, homogenized and grounded into powder. Then
149 sample (1 g) was weighed into a 12 mL glass tube and supplemented with 100 µL of a methanol
150 solution of BP-d10 (250 ng/mL). The sample was subjected to sonication with 7 mL of methanol
151 (0.5% v/v, formic acid) as extraction solvent in a bath for 5 min and centrifuged for 5 min (4050
152 × g). The extraction procedure was repeated three times, and the supernatants were combined.
153 To remove interferences from the matrix, a clean-up procedure based on dispersive solid
154 phase extraction (d-SPE) was performed by adding 0.3 g of C18 sorbent. The tubes were shaken
155 vigorously for 2 min and then centrifugated at 4050× *g* for 5 min. The supernatant was
156 evaporated to dryness under a nitrogen stream, reconstituted in 0.25 mL of a mixture
157 methanol:water (50:50 v/v) and filtered through a 0.22 µm nylon filter. A 10 µL aliquot was
158 injected into the liquid chromatography tandem mass spectrometry (LC-MS/MS) instrument.

## 159 Liquid chromatography–tandem mass spectrometry conditions

160 Chromatographic analyses were performed using an Agilent 1260 Infinity II (Agilent,
161 Santa Clara, CA, USA) according to method described by Martín et al. (2023). Separation was
162 made up with a HALO C18 Rapid Resolution (50 x 4.6 mm i.d., 2.7 µm) (Teknokroma,
163 Barcelona, Spain). The mobile phase was composed of methanol (solvent A) and a buffer
164 solution acetic acid/ammonium acetate (pH 4.4) (solvent B). The elution program was as
165 follows: 0-14 min, linear gradient from 28 to 70% of solvent A, increased to 80% of A in 5 min
166 and to 100% of A in 6 min and held for 2 min. Flow rate was 0.6 mL/min.
167 The LC system was coupled to a 6495 triple quadrupole mass spectrometer with
168 electrospray ionization source operated in negative mode. Two multiple reaction monitoring
169 (MRM) transitions, for identification and quantification purposes, were selected for each
170 benzophenone studied. Instrument settings and analytical determination parameters are
171 summarized in the [Supplementary Material (Table S1](https://www.ncbi.nlm.nih.gov/pmc/articles/PMC9916121/#app1-ijerph-20-01928)).

## 172 Validation requirements

173 The analytical features (linearity, sensitivity and accuracy (trueness and precision)) of
174 the method are presented in Table S[2.](https://www.ncbi.nlm.nih.gov/pmc/articles/PMC9916121/table/ijerph-20-01928-t001/)
175 A matrix-matched calibration was performed for quantification purposes and to correct
176 matrix effect. To this end, commercial guano samples were fortified with the analytes at eight
177 different concentration levels in the range from method quantitation limit (MQL) to 100 ng/g
178 dw (0.01, 0.05, 0.25, 1.00, 5.00, 25.0, 50.0 and 100 ng/g dw). The commercial guano samples
179 used for matrix-matched calibration were simultaneously analyzed. BPs were not detected in
180 the commercial guano samples.
181 To guarantee the quality assurance of results, a protocol involving the use of control
182 spiked samples including fortified commercial guano samples (25 ng/g dw), solvent, standards
183 containing a mixture of the target compounds in pure solvent (100 ng/mL) and procedural
184 blanks (without guano, processed in the same way as the samples) injections were included into
185 each analytical batch (5 samples).

## 186 Statistical Analysis

187 The statistical analysis of the differences in BP-1 and BP-3 concentration levels of
188 particular bat colonies was performed using GraphPad Prism version 9.2.0 (GraphPad
189 Software, San Diego, CA USA) and a Kruskal–Wallis test was used. Differences were
190 considered statistically significant at *p* < 0.05.
191 **Results**
192 During the present investigations, BP-1 was found in all samples included in the study
193 (Table S3). Furthermore, its concentration levels were the highest among the substances tested
194 (Table 2).
195 Table 2 [Concentration values](https://www.sciencedirect.com/topics/earth-and-planetary-sciences/concentration-value) (ng/g) and frequency of detection (%) of benzophenones in the
196 analyzed guano samples (n = 40)—cumulative data.

| Compound Range | | Arithmetic | Geometric Median Frequency of Detection | | |
| --- | --- | --- | --- | --- | --- |
|  | (ng/g) | Mean | Mean |  | above MQL (%) |
| BP-1 | <MQL-259 | 41.50 | 27.24 | 34.8 | 97.5 |
| BP-2 | <MDL | - | - | - | 0 |
| BP-3 | <MQL-19 | 6.67 | 3.83 | 5.05 | 40 |
| BP-8 | <MDL | - | - | - | 0 |

197 Compound acronyms: BP-1: benzophenone 1; BP-2: benzophenone 2; BP-3: benzophenone 3;
198 BP-8: benzophenone 8; MQL—Method Quantification Limit ((0.10 ng/g dw); MDL - Method
199 Detection Limit (0.04 ng/g dw)
200 BP-1 concentration levels fluctuated from <0.1 ng/g to 259 ng/g with a mean value of
201 41.50 ng/g. BP-1 in concentration levels above MQL was observed in 97.5% samples included
202 in the study (Table S3). The second most common BP was BP-3, which concentration levels
203 higher than MQL were observed in 40% of the samples included in this investigation (Table 2).
204 BP-3 concentration levels fluctuated from <0.1 ng/g to 19 ng/g with a mean value of 6.67 ng/g.
205 Clear differences in the concentration levels of BP-1 and BP-3 were visible between particular
206 samples, even in those that were collected from the same colony. The most visible differences
207 in BPs concentration levels in guano samples within one colony were noted in the case of BP-
208 1 in the colony no.2, where values fluctuated from 30.5 ng/g to 259 ng/g (Table S3). Contrary
209 to BP-1 and BP-3, the concentration levels of BP-2 and BP-8 did not exceed the method
210 detection limit (MDL) (0.1 ng/g) in any sample included in the study (Table S3).
211 Furthermore, clear differences in BP-1 concentration levels were found between bat
212 colonies included in the study. Higher concentration levels of this substance were found in bat
213 colony no. 2 and no. 3, where the mean values (±SD) amounted to 76.71±65.51 ng/g and
214 56.97±14.42 ng/g, respectively. In colonies no. 1 and no. 3 BP-1 concentration levels were
215 statistically significantly lower than in colonies no. 2 and no. 3 and achieved 18.08±6.81 ng/g
216 (colony no.1) and 11.23±13.13 ng/g (colony no. 4) (Fig. 2A).

13

218 Mean concentration levels of BP-3 amounted to 5.03±6.03 ng/g in colony no. 1,
219 8.02±9.31 ng/g in colony no. 2, 5.76 ng/g in colony no. 3 and 9.18±7.65 ng/g in colony no. 4
220 (Fig 3B). No statistically significant differences were observed between particular colonies in
221 BP-3 concentration levels.
222 **Discussion**
223 This study has shown that wild bats are exposed to BPs, but the comparison of obtained
224 results with previous investigations shows difficulties in interpretation, because the existing
225 research, focusses mainly on humans and aquatic organisms, has been conducted on other
226 matrices (Table 3).
227 Table 3 Selected previous studies on concentration levels of benzophenone 1 (BP-1), benzophenone 2
228 (BP-2), benzophenone 3 (BP-3) and benzophenone 8 (BP-8) in humans and animals. Benzophenone
229 concentration levels are shown in ng/g (in solid matrices) or ng/mL (in liquid matrices)

| Species | Country | Matrix | n | BP-1 | BP-2 | BP-3 | BP-8 | ref |
| --- | --- | --- | --- | --- | --- | --- | --- | --- |
| Humans | Australia | urine | 24 |  |  | 16.5-312 |  | Heffernan et al.  2015 |
|  | China | Amniotic  fluid | 15 | <0.02–3.22 |  | <0.01–0.38 | <0.01–0.60 | Song et al.,  2020 |
|  | Brazil | urine | 300 | 0.01-1910 | 0.25-8.25 | 0.7-9.83 | 0.01-2.69 | Rocha et al.  2018 |
|  | China | urine | 100 | <0.07-14.6 |  | <0.11-46.1 |  | Zhang et al.  2013 |
|  |  | blood | 75 | <0.06-0.15 |  | <0.41-3.88 |  |  |
|  | Denmark | urine | 309 | <LOD-129 | <LOD-9.84 | <LOD-644 |  | Frederiksen et al. 2021 |
|  |  | Blood  serum | 306 | <LOD-0.86 | <LOD-0.10 | <LOD-5.18 |  |  |
|  |  | Seminal  plasma | 297 | <LOD-10.9 | <LOD | <LOD-5.94 |  |  |
|  | Spain | Breast milk | 10 | ND-0.51 |  | ND-15.7 | ND-0.73 | Rodríguez- Gómez et al.  2015 |
|  |  | placenta | 79 | <LOD-3.32 | 0.38-1.93 | 0.65-3.09 | <LOD | Fernández et al.,  2016 |
|  |  | nails | 22 | 6.3-194 | ND | 25-245 | ND-3.9 | Martin-Pozo et  al., 2021 |
|  | USA | urine | 2517 |  |  | 0.4-21700 |  | Calafat et al.  2008 |
| Dogs | USA | urine | 50 | <LOQ-9.2 |  | <LOQ-46 | LOQ-0.7 | Karthikraj et al.  2020 |
| Cats | USA | urine | 50 | <LOQ-13.5 |  | <LOQ-61.6 | <LOQ-8.4 |  |
| Wild bats | Poland | guano | 5 | 10.5-34.8 | <LOD | <LOQ-15.5 | <LOD | Martin et al.,  2023 |
| Beluga  whale | Canada | liver | 80 |  |  | <LOD-1320 |  | Blouin et al.  2022 |
| Wild birds | Spain | eggs |  | <LOQ-677 |  | 16.9-49.3 |  | Molins-Delgado  et al. 2017 |

| Turtles | Italy | blood | 32 |  |  | <LOD-  28430 |  | Cocci et al.  2020 |
| --- | --- | --- | --- | --- | --- | --- | --- | --- |
| Fish | Brazil | body | 11 | <LOQ |  | 3.5-15.4 |  | Molins-Delgado  et al. 2018b |
|  | Greece | body | 8 | ND | 11.8-41.9 | <LOQ-1.8 |  | Diaz-Cruz et al.  2019 |
|  | Spain | body | 193 |  |  | ND-2.2 |  | Gago Ferrero et  al. 2015 |
|  | Taiwan | body | 4 | 0.7-3.6 |  | 3.3-6.9 | 0.5-2.4 | Tsai et al. 2014 |

230 LOD – limit of detection, LOQ-limit of quantification, ND- not detected
231 Results obtained in the present study also confirms that bat guano is the appropriate
232 matrix to evaluate the exposure of these animals to BPs, as we have observed in our preliminary
233 studies (Martin et al., 2023).
234 [A vast majority of previous papers](https://context.reverso.net/tłumaczenie/angielski-polski/A%2Bvast%2Bmajority%2Bof%2Bpapers) concern human exposure to BPs. Review article
235 written by Mao et al. (2022) mentions above 160 investigations describing BPs concentration
236 levels in humans in various parts of the world. Previous investigations on BPs in humans have
237 mainly described BPs in the urine samples, but these substances have also been found in human
238 blood, breast milk, amniotic fluid and even in the brain (Table 3). The latter proves the ability
239 of BPs to cross the blood-brain barrier. In turn, previous research on BPs in wild animals mainly
240 focuses on bodies of fish and other water organisms, and only a few studies concern terrestrial
241 birds (Table 3).
242 Analyzing previous studies, it is clear that the concentration of BPs in the organisms of
243 humans and animals varies significantly depending on the species studied, the region in which
244 the study was conducted and the type of BP. It is well known that BP concentration levels in
245 the living organisms depend on the presence of BPs in the natural environment, resulting from
246 the urbanization and industrialization of a given area, as well as the frequency of the use of
247 sunscreens and personal care products by the inhabitants (Calafat et al. 2008; Silveira et al.
248 2020; Mao et al. 2022).
249 Much [less is known](https://context.reverso.net/tłumaczenie/angielski-polski/less%2Bis%2Bknown) about interspecies differences in BPs metabolism. The knowledge
250 of this item is practically limited to selected BPs (mainly BP-3) and laboratory animals.
251 Furthermore, the information is not inconclusive. That is, some older studies have shown
252 differences in BP-3 metabolism between rats and mice (Okere and Abdel-Rhaman 1994; Okere
253 et al. 1994; Kadry et al. 1995), while more recent research has found that generally BP-3
254 metabolism in rats and mice is similar with only minor differences (Mutlu et al. 2019).
255 Given all the above, the comparison of the results obtained in the present study with
256 previous investigation is rather difficult. However, this investigation has shown that all bat
257 colonies included in the study are exposed to BPs, which strongly suggests the environmental
258 pollution with these substances in Poland. Furthermore, the results indicate that the greatest
259 threat to wild bats are BP-1 and BP-3. It is in agreement with most previous studies, in which
260 these compounds have been found in humans and animals in the highest amounts among BPs
261 (Table 3). Relatively high BP-1 concentration levels observed both in the present investigation
262 and previous studies may result not only from environmental pollution with this compound, but
263 also from the fact that BP-1 is one of the metabolites of BP-3 and can be formed in living
264 organisms after absorption of BP-3 (Nakagawa et al. 2002).
265 Despite the differences in the matrices used between the current study and previous
266 investigations, it can be concluded that the exposure of wild bats to BP-1 and BP-3 is relatively
267 high. Concentration levels of BP-1 and BP-3 noted in this research are higher not only from
268 those noted in terrestrial birds and most of marine organisms, but also from those noted in some
269 studies conducted on humans (Table 3). The latter fact is quite surprising, since BPs are
270 anthropogenic pollutants, and humans are significantly exposed to BPs using sunscreens and
271 personal care products containing these compounds (Mao et al. 2022). Relatively high
272 concentration levels of BPs in bats may result from the fact that the colonies are located in the
273 immediate vicinity of human settlements (Russo and Ancillotto 2015), and therefore the animals
274 can be exposed to anthropogenic pollutants to a large extent. However, it cannot be ruled out
275 that higher concentration levels noted in this study result from the character of the matrix used.
276 Namely, at least part of the BPs found in guano samples are substances that were present in
277 water or food and were not absorbed through the digestive tract.
278 It should be pointed out that until now feces/guano samples have not been used to assess
279 human and animal exposure to BPs. It is connected with the fact that, in light of previous
280 studies, the main route of elimination of BPs from living organisms is urinary excretion (Okere
281 and Abdel-Rhaman 1994; Kadry et al. 1995; Mutlu et al. 2019), and therefore most studies on
282 humans have described BPs in urine samples (Mao et al. 2022). However, it is known that BPs
283 may be eliminated from organisms also with feces. Previous studies on [C^14^]BP-3 in
284 experimental rodents have found that from 15% to 42% of this substance (depending on the
285 route of exposure and animal species) may be excreted with the feces (Dareer et al. 1986; Mutlu
286 et al. 2019). For this reason, the analysis of feces/guano samples seems to be an appropriate
287 method to assess the degree of exposure of living organisms to BPs, which is confirmed by the
288 results of this study. The usefulness of guano samples for determining bat exposure to BPs was
289 also demonstrated by our earlier preliminary publication performed on very limited number of
290 indyviduals (n=5) (Martin etr al., 2023).
291 Feces/guano analysis is particularly important for studies concerning wild protected
292 animals. Contrary to collection of other types of samples (blood, urine, tissues, hair), which
293 [require](https://context.reverso.net/tłumaczenie/angielski-polski/require)s animals [to be captured or killed,](https://context.reverso.net/tłumaczenie/angielski-polski/to%2Bbe%2Bkilled) feces samples may be taken without significantly
294 interfering with animal life. Of course feces/guano analysis also has its drawbacks. During
295 studies, it is often impossible to assign a sample to a concrete animal, and therefore it cannot
296 be determined its age, sex, and health status, and it is known that these parameters often affect
297 the degree of exposure to environmental pollutants (Bowman et al. 2014; Forthun et al. 2023).
298 During feces/guano samples analysis attention should also be paid to taking fresh samples,
299 because in samples remaining for a long time outside the animal's body, either the
300 decomposition of the given substance may occur, or the sample may be contaminated with
301 substances from the external environment. Moreover, as mentioned above, feces/guano samples
302 analysis evaluates not only part of substance, which was absorbed into the body and then
303 excreted with feces, but also that which was in the food and drinking water, but was not
304 absorbed. However, analysis of feces/guano samples are a good alternative to “classical”
305 matrices in BPs biomonitoring in wild animals, what is confirmed by the present study.
306 During the present study statistically significant differences have been observed
307 between particular bat colonies in the degree of BP-1 concentration levels in guano samples. It
308 is in agreement with previous studies, which have found that BPs levels in the natural
309 environment clearly depend on the region, where research has been conducted (Mao et al.
310 2022). The differences noted in the present study do not appear to depend only on the degree
311 of industrialization and urbanization of the area. However, the second high concentration level
312 of BP-1 was observed in colony no. 3 located in Pulawy - relatively big city (47417 inhabitants)
313 with chemical industry, but highest levels of BP-1 has been found in colony no. 2 located in
314 small village (2464 inhabitants) without any industrial plants. In the latter case, high levels of
315 BP-1 in bat guano samples may be related to the use of BPs in agriculture (Hong et al. 2021).
316 However, the results obtained have shown that bat exposure to BPs is affected by some
317 unidentified factors that are difficult to determine without comprehensive environmental and
318 demographic studies (for example, investigations with regard to sunscreen, cosmetics, and
319 personal care products consumption).
320 Of course, the [question also arises](https://context.reverso.net/tłumaczenie/angielski-polski/question%2Barises) whether BPs concentration levels noted in guano
321 samples during the present study may have a negative impact on bat health status. The answer
322 is rather difficult. In light of previous studies, it is relatively well known that BPs affect the
323 functions of many internal organs, influencing, among others, the endocrine, reproductive, and
324 neuronal systems. Furthermore, in studies conducted on wild animals, clear positive
325 correlations have been found between BP levels and other active sunscreen ingredients and
326 gene biomarkers of inflammation, oxidative stress, and hormonal activity (Cocci et al. 2020).
327 On the other hand, metabolism, [toxicokinetic](https://context.reverso.net/tłumaczenie/angielski-polski/Toxicokinetics) tissue distribution and therefore adverse effects
328 of BPs depend on the animal species (Kadry et al. 1995; Mutlu et al. 2019), and until now these
329 aspects in bats have not been studied. Due to the fact that this is the first study on BP levels in
330 guano samples, the correlations between the levels of BPs in feces/guano and internal organs
331 of animals are unknown. However, given that BPs can also affect organisms at low doses
332 (Matouskova et al. 2020), and wild animals are often exposed to a variety of synergistic
333 environmental pollutants (Iatrou et al. 2019), it can be assumed that the levels of BP observed
334 in this study may negatively affect bat health status. Nonetheless further research on this topic
335 is needed.

## 336 Conclusion

337 The present work is the first study focuses on the assessment of wild terrestrial mammals
338 to BPs through the analysis of guano/feces samples. The results obtained have demonstrated
339 that wild bats are exposed to BP-1 and BP-3, which confirms that the natural environment is
340 contaminated with these substances. These observations also strongly suggest that BPs
341 polluting the environment may affect the health status of wild terrestrial mammals. In light of
342 the present study, bats also seem to be a good bioindicator for monitoring BPs in the
343 environment.
344 Furthermore, the present study has shown that feces/guano samples, despite some
345 limitations, are a good alternative to urine or blood samples in the assessment of the wild animal
346 exposure to BPs. Feces/guano samples are of particular importance in the case of research on
347 protected wild animals, because collection of such samples is easy and does not require (unlike
348 other matrices) capturing animals. However, due to intraspecies differences between BPs
349 metabolism and toxicinetics, and the lack of knowledge about the correlation between the
350 presence of BPs in the guano and their distribution in internal organs, it is difficult to accurately
351 assess what levels of BPs in guano [may be a sign of](https://context.reverso.net/tłumaczenie/angielski-polski/may%2Bbe%2Ba%2Bsign%2Bof) the degree of exposure to these substances,
352 which affects the animal health status. Therefore, further studies on this subject are needed.

## 353 References

354 Aparicio I, Martín J, Abril C, Santos JL, Alonso E. Determination of household and industrial
355 chemicals, personal care products and hormones in leafy and root vegetables by liquid
356 chromatography-tandem mass spectrometry*.* J Chromatogr A. 2018;1533:49-56.

357

358 Balmer ME, Buser HR, Müller MD, Poiger T. Occurrence of some organic UV filters in
359 wastewater, in surface waters, and in fish from Swiss Lakes. Environ Sci Technol. 2005;39:953-
360 62.

361

362 Blouin K, Malaisé F, Verreault J, Lair S, Lu Z. Occurrence and temporal trends of industrial
363 antioxidants and UV absorbents in the endangered St. Lawrence Estuary beluga whale
364 (Delphinapterus leucas). Sci Total Environ. 2022;842:156635.

365

366 Bowman RE, Luine V, Khandaker H, Villafane JJ, Frankfurt M. Adolescent bisphenol-A
367 exposure decreases dendritic spine density: role of sex and age. Synapse. 2014;68:498-507.

368

369 Buck Louis GM, Maisog J, Sapra KJ, Maisog J, Sundaram R. Urinary concentrations of
370 benzophenone-type ultraviolet radiation filters and couples’ fecundity. Am J Epidemiol.
371 2014;180: 1168-75.

372

373 Calafat AM, Wong LY, Ye X, Reidy JA, Needham LL. Concentrations of the sunscreen agent
374 benzophenone-3 in residents of the United States: National Health and Nutrition Examination
375 Survey 2003--2004. Environ Health Perspect. 2008;116:893-7.

376

377 Camino-Sánchez FJ, Zafra-Gómez A, Dorival-García N, Juárez-Jiménez B, Vílchez JL.
378 Determination of selected parabens, benzophenones, triclosan and triclocarban in agricultural
379 soils after and before treatment with compost from sewage sludge: A lixiviation study. Talanta.
380 2016;150:415-24.
381 Careghini A, Mastorgio AF, Saponaro S, Sezenna E. Bisphenol A, nonylphenols,
382 benzophenones, and benzotriazoles in soils, groundwater, surface water, sediments, and food:
383 a review*.* Environ Sci Pollut Res Int. 2015;22:5711-41.

384

385 Castro G, Fourie AJ, Marlin D, Venkatraman V, González SV, Asimakopoulos AG. Occurrence
386 of bisphenols and benzophenone UV filters in wild brown mussels (Perna perna) from Algoa
387 Bay in South Africa. Sci Total Environ. 2022;813:152571.

388

389 Cocci P, Mosconi G, Palermo FA. Sunscreen active ingredients in loggerhead turtles (Caretta
390 caretta) and their relation to molecular markers of inflammation, oxidative stress and hormonal
391 activity in wild populations. Mar Pollut Bull. 2020;153:111012.

392

393 Dareer SM, Kalin JR, Tillery KF, Hill DL. Disposition of 2-hydoxy-4 methoxybenzophenone
394 in rats dosed orally, intravenously, or topically. J Toxicol Environ Health. 1986;19:491–502.

395

396 Díaz-Cruz MS, Molins-Delgado D, Serra-Roig MP, Kalogianni E, Skoulikidis NT, Barceló D.
397 Personal care products reconnaissance in EVROTAS river (Greece): Water-sediment partition
398 and bioaccumulation in fish. Sci Total Environ. 2019;651:3079-3089.

399

400 Dulsat-Masvidal M, Ciudad C, Infante O, Mateo R, Lacorte S. Water pollution threats in
401 important bird and biodiversity areas from Spain. J Hazard Mater. 2023;448:130938.

402

403	Emnet P, Mahaliyana AS, Northcott G, Gaw S. Organic micropollutants in wastewater effluents

404	and the receiving coastal waters, sediments, and biota of Lyttelton Harbour (Te Whakaraupō),

405	New Zealand. Arch Environ Contam Toxicol. 2020;79:461-477.

406

407	Forthun IH, Roelants M, Haug LS, Knutsen HK, Schell LM, Jugessur A, et al. Levels of per-

408	and polyfluoroalkyl substances (PFAS) in Norwegian children stratified by age and sex - Data

409	from the Bergen Growth Study 2. Int J Hyg Environ Health. 2023;252:114199.

410

411	Foubert K, Dendooven E, Theunis M, Naessens T, Ivanova B, Pieters L, et al. The presence of

412	benzophenone in sunscreens and cosmetics containing the organic UV filter octocrylene: A

413	laboratory study. Contact Dermatitis. 2021;85:69-77.

414

415	Frederiksen H, Krause M, Jørgensen N, Rehfeld A, Skakkebæk NE, Andersson AM. UV filters

416	in matched seminal fluid-, urine-, and serum samples from young men. J. Expo. Sci. Environ.

417	Epidemiol. 2021;31:345-355.

418

419	Gago-Ferrero P, Díaz-Cruz MS, Barceló D. UV filters bioaccumulation in fish from Iberian

420	river basins. Sci Total Environ. 2015;518-519:518-25.

421

422	Gavrila AA, Dasteridis IS, Tzimas AA, Chatzimitakos TG, Stalikas CD. Benzophenones in the

423	environment: Occurrence, fate and sample preparation in the analysis. Molecules.

424	2023;28:1229.

425

426	González-Rubio S, Vike-Jonas K, Gonzalez SV, Ballesteros-Gómez A, Sonne C, Dietz R, et al.

427	Bioaccumulation potential of bisphenols and benzophenone UV filters: A multiresidue

428	approach in raptor tissues. Sci Total Environ. 2020;741:140330.

429

430	Heffernan AL, Baduel C, Toms LM, Calafat AM, Ye X, Hobson P, et al. Use of pooled samples

431	to assess human exposure to parabens, benzophenone-3 and triclosan in Queensland, Australia.

432	Environ Int. 2015;85:77-83.

433

434	Hewitt NJ, Grégoire S, Cubberley R, Duplan H, Eilstein J, Ellison C, et al. Measurement of the

435	penetration of 56 cosmetic relevant chemicals into and through human skin using a standardized

436	protocol. J Appl Toxicol. 2020;40:403-415.

437

438	Holmboe SA, Scheutz Henriksen L, Frederiksen H, Andersson AM, Priskorn L, Jørgensen N,

439	et al. Prenatal exposure to phenols and benzophenones in relation to markers of male

440	reproductive function in adulthood. Front Endocrinol (Lausanne). 2022;13:1071761.

441

442	Hong M, Yang X, Zhang X, Ji Y, Zhou L, Xiu G, et al. Aqueous photodegradation of the

443	benzophenone fungicide metrafenone: Carbon-bromine bond cleavage mechanism. Water Res.

444	2021;206:117775.

445

446	Iatrou EI, Tsygankov V, Seryodkin I, Tzatzarakis MN, Vakonaki E, Barbounis E, et al.

447	Monitoring of environmental persistent organic pollutants in hair samples collected from wild

448	terrestrial mammals of Primorsky Krai, Russia. Environ Sci Pollut Res Int. 2019;26:7640-7650.

449

450	Kadry AM, Okereke CS, Abdel-Rahman MS, Friedman MA, Davis RA. Pharmacokinetics of

451	benzophenone-3 after oral exposure in male rats. J Appl Toxicol. 1995;15:97-102.

452

453	Karthikraj R, Lee S, Kannan K. Biomonitoring of exposure to bisphenols, benzophenones,

454	triclosan, and triclocarban in pet dogs and cats. Environ Res. 2020;180:108821.

455

456	Kim S, Choi K. Occurrences, toxicities, and ecological risks of benzophenone-3, a common

457	component of organic sunscreen products: a mini-review. Environ Int. 2014;70:143-57.

458

459	Kunisue T, Chen Z, Buck Louis GM, Sundaram R, Hediger ML, Sun L, Kannan K. Urinary

460	concentrations of benzopheone-type UV filters in U.S. women and their association with

461	endometriosis. Environ Sci Technol. 2012;46:4624-32.

462

463	Li J, Lam JCW, Li W, Du B, Chen H, Zeng L. Occurrence and distribution of photoinitiator

464	additives in paired maternal and cord plasma in a South China population. Environ Sci Technol.

465	2019;53:10969-10977.

466

467	Liao C, Kannan K. Widespread occurrence of benzophenone-type UV light filters in personal

468	care products from China and the United States: an assessment of human exposure. Environ Sci

469	Technol. 2014;48:4103-9.

470

471	Liao C, Kannan K. Species-specific accumulation and temporal trends of bisphenols and

472	benzophenones in mollusks from the Chinese Bohai Sea during 2006-2015. Sci Total Environ.

473	2019;653:168-175.

474

475	Ma B, Lu G, Liu F, Nie Y, Zhang Z, Li Y. Organic UV filters in the surface water of Nanjing,

476	China: Occurrence, distribution and ecological risk assessment. Bull Environ Contam Toxicol.

477	2016;96:530-5.

478

479	Mao F, He Y, Gin KYH. Occurrence and fate of benzophenone-type UV filters in aquatic

480	environments: A review. Environ Sci Water Res Technol. 2019;5:209–223.

481

482	Mao JF, Li W, Ong CN, He Y, Jong MC, Gin KY. Assessment of human exposure to

483	benzophenone-type UV filters: A review. Environ Int. 2022;167:107405.

484

485	Martin J, Gonkowski S, Kortas A, Sobiech P, Rytel L, Santos J, et al. Multiclass method to

486	determine emerging pollutants in bats using a non-invasive approach based on guano matrix.

487	Microchem J. 2023;188:108486.

488

489	Matouskova K, Jerry DJ, Vandenberg LN. Exposure to low doses of oxybenzone during

490	perinatal development alters mammary gland morphology in male and female mice. Reprod

491	Toxicol. 2020;92:66-77.

492

493	Molins-Delgado D, Máñez M, Andreu A, Hiraldo F, Eljarrat E, Barceló D, et al. A potential new

494	threat to wild life: Presence of UV filters in bird eggs from a preserved area. Environ Sci

495	Technol. 2017;51:10983-10990.

496

497	Molins-Delgado D, Olmo-Campos MDM, Valeta-Juan G, Pleguezuelos-Hernández V, Barceló

498	D, Díaz-Cruz MS. Determination of UV filters in human breast milk using turbulent flow

499	chromatography and babies' daily intake estimation. Environ Res. 2018ª;161:532-539.

500

501	Molins-Delgado D, Muñoz R, Nogueira S, Alonso MB, Torres JP, Malm O, et al. Occurrence

502	of organic UV filters and metabolites in lebranche mullet (Mugil liza) from Brazil. Sci Total

503	Environ. 2018;618:451-459.

504

505	Mutlu E, Garner CE, Wegerski CJ, McDonald JD, McIntyre BS, Doyle-Eisele M, et al.

506	Metabolism and disposition of 2-hydroxy-4-methoxybenzophenone, a sunscreen ingredient, in

507	Harlan Sprague Dawley rats and B6C3F1/N mice; a species and route comparison. Xenobiotica.

508	2020;50:689-704.

509

510	Nakagawa	Y,	Suzuki	T.	Metabolism	of	2-hydroxy-4-methoxyben-zophenone	in

511	isolated rat hepatocytes and xenoestrogenic effects of its me-tabolites on MCF-7 human

512	breast cancer cells. Chem Biol Interact. 2002;139:115-28.

513

514	Nakamura N, Inselman AL, White GA, Chang CW, Trbojevich RA, Sephr E, et al. Effects of

515	maternal and lactational exposure to 2-hydroxy-4-methoxybenzone on development and

516	reproductive organs in male and female rat offspring. Birth Defects Res B Dev Reprod Toxicol*.*

517	2015;104:35-51.

518

519	Okereke CS, Abdel-Rhaman MS. Species differences in the disposition of benzophenone-3

520	after oral administration in rat and mouse. Toxic Subst J. 1994;13:239–251.

521

522	Oró-Nolla B, Lacorte S, Vike-Jonas K, Gonzalez SV, Nygård T, Asimakopoulos AG, et al.

523	Occurrence of bisphenols and benzophenone UV filters in white-tailed eagles (*Haliaeetus*

524	*albicilla*) from Smøla, Norway. Toxics. 2021;9:34.

525

526	Rocha BA, Asimakopoulos AG, Honda M, da Costa NL, Barbosa RM, Barbosa F Jr., et al.

527	Advanced data mining approaches in the assessment of urinary concentrations of bisphenols,

528	chlorophenols, parabens and benzophenones in Brazilian children and their association to DNA

529	damage. Environ Int. 2018;116:269-277.

530

531	Rodríguez-Gómez R, Zafra-Gómez A, Dorival-García N, Ballesteros O, Navalón A.

532	Determination of benzophenone-UV filters in human milk samples using ultrasound-assisted

533	extraction and clean-up with dispersive sorbents followed by UHPLC-MS/MS analysis.

534	Talanta. 2015;134:657-664.

535

536	Russo D, Ancillotto L. Sensitivity of bats to urbanization: A review. Mamm Biol. 2015;80:205–

537	212.

538

539	Russo D, Salinas-Ramos VB, Cistrone L, Smeraldo S, Bosso L, Ancillotto L. Do we need to

540	use bats as bioindicators? Biology. 2021;10:693.

541

542	Sang Z, Leung KS. Environmental occurrence and ecological risk assessment of organic UV

543	filters in marine organisms from Hong Kong coastal waters. Sci Total Environ. 2016;566-

544	567:489-498.

545

546	Santamaría CG, Abud JE, Porporato MM, Meyer N, Zenclussen AC, Kass L, et al. The UV

547	filter benzophenone 3, alters early follicular assembly in rat whole ovary cultures. Toxicol Lett.

548	2019;303:48-54.

549

550	Schneider SL, Lim HW. Review of environmental effects of oxybenzone and other sunscreen

551	active ingredients. J Am Acad Dermatol. 2019;80:266-271.

552

553	Shin JC, Lee E, An S, Jin SH, Ha J, Choi WJ, et al. Benzophenone-3 and benzophenone-8

554	exhibit obesogenic activity via peroxisome proliferator-activated receptor γ pathway. Toxicol

555	In Vitro. 2020;67:104886

556

557 Silveira RS, Rocha BA, Rodrigues JL, Barbosa F Jr. Rapid, sensitive and simultaneous
558 determination of 16 endocrine-disrupting chemicals (parabens, benzophenones, bisphenols, and
559 triclocarban) in human urine based on microextraction by packed sorbent combined with liquid
560 chromatography	tandem	mass	spectrometry	(MEPS-LC-MS/MS).	Chemosphere.
561 2020;240:124951.

562

563	Song S, He Y, Huang Y, Huang X, Guo Y, Zhu H, et al. Occurrence and transfer of

564	benzophenone-type ultraviolet filters from the pregnant women to fetuses. Sci Total Environ.

565	2020;726:138503.

566

567	Sotero DF, Benvindo-Souza M, Pereira de Freitas R, de Melo E, Silva D. Bats and pollution:

568	Genetic approaches in ecotoxicology. Chemosphere. 2022;307:135934.

569

570	Sun H, Li Y, Huang C, Peng J, Yang J, Sun X, et al. Solid-phase extraction based on a

571	molecularly imprinted polymer for the selective determination of four benzophenones in tap

572	and river water. J Sep Sci. 2015;38:3412-20.

573

574	Tang Z, Han X, Li G, Tian S, Yang Y, Zhong F, et al. Occurrence, distribution and ecological

575	risk of ultraviolet absorbents in water and sediment from Lake Chaohu and its inflowing rivers,

576	China. Ecotoxicol Environ Saf. 2018;164:540-547.

577

578	Tsai DY, Chen CL, Ding WH. Optimization of matrix solid-phase dispersion for the rapid

579	determination of salicylate and benzophenone-type UV absorbing substances in marketed fish.

580	Food Chem. 2014;154:211-6.

581

582	van der Meer TP, Artacho-Cordón F, Swaab DF, Struik D, Makris KC, Wolffenbuttel BHR, et

583	al. Distribution of non-persistent endocrine disruptors in two different regions of the human

584	brain. Int J Environ Res Public Health. 2017;14:1059.

585

586	Vela-Soria F, Jiménez-Díaz I, Rodríguez-Gómez R, Zafra-Gómez A, Ballesteros O, Navalón A,

587	et al. Determination of benzophenones in human placental tissue samples by liquid

588	chromatography-tandem mass spectrometry. Talanta. 2011;85:1848-55.

589

590	Vila M, Lamas JP, Garcia-Jares C, Dagnac T, Llompart M. Optimization of an analytical

591	methodology for the simultaneous determination of different classes of ultraviolet filters in

592	cosmetics by pressurized liquid extraction-gas chromatography tandem mass spectrometry. J

593	Chromatogr A. 2015;1405:12-22.

594

595	Wan Y, Xue J, Kannan K. Occurrence of benzophenone-3 in indoor air from Albany, New York,

596	USA, and its implications for inhalation exposure. Sci Total Environ. 2015;537:304-8.

597

598	Wang S, Huo Z, Shi W, Wang H, Xu G. Urinary benzophenones and synthetic progestin in

599	Chinese adults and children: concentration, source and exposure. Environ Sci Pollut Res Int.

600	2021;28:50245-50254.

601

602	Wilson DE, Mittermeier RA. Handbook of the mammals of the world–volume 9 Bats. Linx,

603	editor. Assoc. Conserv. Int. IUCN, Barcelona. 2019.

604

605	Wnuk A, Rzemieniec J, Staroń J, Litwa E, Lasoń W, Bojarski A, et al. Prenatal exposure to

606	benzophenone-3 impairs autophagy, disrupts RXRs/PPARγ signaling, and alters epigenetic and

607	post-translational statuses in brain neurons. Mol Neurobiol. 2019;56:4820-4837.

608

609	Wnuk W, Michalska K, Krupa A, Pawlak K. Benzophenone-3, a chemical UV-filter in

610	cosmetics: is it really safe for children and pregnant women? Postepy Dermatol Alergol.

611	2022;39:26-33.

612

613	Wu SB, Long C, Kennelly EJ. Structural diversity and bioactivities of natural benzophenones.

614	Nat Prod Rep. 2014;31:1158-74.

615

616	Xue J, Liu W, Kannan K. Bisphenols, benzophenones, and bisphenol A diglycidyl ethers in

617	textiles and infant clothing. Environ Sci Technol. 2017;51:5279-5286.

618

619	Zhang Z, Ren N, Li YF, Kunisue T, Gao D, Kannan K. Determination of benzotriazole and

620	benzophenone UV filters in sediment and sewage sludge. Environ Sci Technol. 2011;45:3909–

621	16.

622

623	Zhang T, Sun H, Qin X, Wu Q, Zhang Y, Ma J, et al. Benzophenone-type UV filters in urine

624	and blood from children, adults, and pregnant women in China: partitioning between blood and

625	urine as well as maternal and fetal cord blood. Sci Total Environ. 2013;461-462:49-55.

626

## 627 Ethical Approval

628 Not applicable. Due to the fact that sampling was not invasive to animals, [in view of](https://context.reverso.net/tłumaczenie/angielski-polski/in%2Bview%2Bof%2Bthe%2Blaw) the Act
629 for the Protection of Animals for Scientific or Educational Purposes of 15 January 2015
630 (Official Gazette 2015, No. 266), applicable in the Republic of Poland, the agreement of Ethical
631 Committee was not required during this investigation.

## 632 Consent to Participate

633 Not applicable**.**

## 634 Consent to Publish

635 Not applicable.

## 636 Authors Contributions

637 Conceptualization was performed by Slawomir Gonkowski and Liliana Rytel. Methodology
638 was performed by Julia Martin, Irena Aparicio, Juan Luis Santos and Esteban Alonso. Sample
639 collection was performed by Liliana Rytel. The first draft of the manuscript was written by
640 Slawomir Gonkowski, review and editing was performed by Andrzej Pomianowski and László
641 Könyves. All authors commented on previous versions of the manuscript. All authors read and
642 approved the final manuscript.

## 643 Funding

644 This project was financially supported by statutory grant of Department of Clinical Physiology,
645 faculty of Veterinary Medicine, University of Warmia and Mazury, Olsztyn, Poland – grant no.
646 15.610.003-110.

## 647 Competing Interests

648 The authors have no relevant financial or non-financial interests to disclose.

## 649 Availability of data and materials

650 All data are presented in the manuscript

651

652 **Figure 1** Localization on bat colonies included into the study
653 **Figure 2** Mean concentration levels (±SD) of A) benzophenone 1 (BP-1) and B) benzophenone
654 3 (BP-3) in particular bat colonies included into the study. Statistically values showing
655 statistically significant differences (at *p* < 0.05) were marked with different letters, values not
656 showing such differences - with the same letters.

Figure 1	[Click here to access/download;Figure;Fig 1(2).jpg](https://www.editorialmanager.com/pone/download.aspx?id=35060531&guid=d3e76046-9895-4890-9772-db7f1d0c0d3a&scheme=1)[<https://www.editorialmanager.com/pone/download.aspx?id=35060531&guid=d3e76046-9895-4890-9772-db7f1d0c0d3a&scheme=1>](https://www.editorialmanager.com/pone/download.aspx?id=35060531&guid=d3e76046-9895-4890-9772-db7f1d0c0d3a&scheme=1)

Figure 2	[Click here to access/download;Figure;Fig 2(1).jpg](https://www.editorialmanager.com/pone/download.aspx?id=35060533&guid=05f818e3-16a6-47ab-858c-09c943848b5e&scheme=1)[<https://www.editorialmanager.com/pone/download.aspx?id=35060533&guid=05f818e3-16a6-47ab-858c-09c943848b5e&scheme=1>](https://www.editorialmanager.com/pone/download.aspx?id=35060533&guid=05f818e3-16a6-47ab-858c-09c943848b5e&scheme=1)

Supporting Information

# [Click here to access/download](https://www.editorialmanager.com/pone/download.aspx?id=35060534&guid=6e5da64b-55c4-49ff-8ebe-7a7b558165c2&scheme=1)

[Supporting Information SUPPLEMENTARY MATERIALS(1).docx](https://www.editorialmanager.com/pone/download.aspx?id=35060534&guid=6e5da64b-55c4-49ff-8ebe-7a7b558165c2&scheme=1)
